# Focalization Performance Study of a Novel Bulk Acoustic Wave Device

**DOI:** 10.3390/nano11102630

**Published:** 2021-10-06

**Authors:** Federica Barbaresco, Luisa Racca, Luca Spigarelli, Matteo Cocuzza, Simone Luigi Marasso, Candido Fabrizio Pirri, Giancarlo Canavese

**Affiliations:** 1 Chilab—Materials and Microsystems Laboratory, DISAT, Politecnico di Torino, Chivasso, 10034 Turin, Italy; matteo.cocuzza@infm.polito.it (M.C.); simone.marasso@polito.it (S.L.M.); fabrizio.pirri@polito.it (C.F.P.); 2 Department of Applied Science and Technology, Politecnico di Torino, Corso Duca degli Abruzzi 24, 10129 Turin, Italy; luisa.racca@polito.it (L.R.); luca.spigarelli@polito.it (L.S.); 3 CNR-IMEM, Parco Area delle Scienze, 37a, 43124 Parma, Italy

**Keywords:** bulk acoustic wave, particle and cell separation, microfluidics, acoustophoresis, transverse path, liquid biopsy

## Abstract

This work illustrates focalization performances of a silicon-based bulk acoustic wave device applied for the separation of specimens owing to micrometric dimensions. Samples are separated in the microfluidic channel by the presence of an acoustic field, which focalizes particles or cells according to their mechanical properties compared to the surrounded medium ones. Design and fabrication processes are reported, followed by focalization performance tests conducted either with synthetic particles or cells. High focalization performances occurred at different microparticle concentrations. In addition, preliminary tests carried out with HL-60 cells highlighted an optimal separation performance at a high flow rate and when cells are mixed with micro and nanoparticles without affecting device focalization capabilities. These encouraging results showed how this bulk acoustic wave device could be exploited to develop a diagnostic tool for early diagnosis or some specific target therapies by separating different kinds of cells or biomarkers possessing different mechanical properties such as shapes, sizes and densities.

## 1. Introduction

Performing an early diagnosis of cancer alterations is of fundamental importance for patients’ clinical evaluation and treatment strategy [1,2]. Generally, conventional tissue biopsy is exploited to assess cancer’s mutational profile (i.e., primary tumor or metastasis), but there are several limitations derived from this approach. Indeed, when it is possible to reach cancer, biopsy sampling involves localized, invasive and harmful surgical interventions [3]. Moreover, throughout tissue biopsy it is not possible to characterize intra- or inter-tumor heterogeneity, a fundamental aspect to assess cancer in its advanced stages or in the presence of different tumor sites. Furthermore, this kind of approach cannot be used to achieve a dynamic follow-up of cancer molecular modifications to evaluate cancer progression and evolutions in patients [4,5]. Thus, to overcome such critical issues, liquid biopsy emerged as a new diagnostic tool to manage lung cancer screening and to adjust therapy according to patients’ personalized treatment [6]. Liquid biopsy concerns the analysis of any tumor-derived material (i.e., circulating tumor cells, exosomes, platelets, tumor-derived nucleic acids, proteins, cytogenetic and cytokinetic parameters) circulating in the blood or any other body fluids instead of a fragment of cancer tissue [7,8,9]. It is a non-invasive and real-time monitoring approach requiring less time and low costs for sample taking. Furthermore, it is a highly sensitive assay able to detect the presence of tumor cells in different organs in patients who lack any clinical or radiological signs or residual tumor cells left behind after local invasive therapy [3,10].

The development of miniaturized laboratory instrumentations and procedures in portable, integrated automated systems in enhanced microfluidic platforms is mandatory to allow a widespread application of liquid biopsy in everyday use either in diagnostic or clinical practice [11,12,13,14]. Acoustophoretic devices among others [15,16,17,18,19,20] emerged in biomedical research, as well as in clinical and diagnostic fields [21,22], for the separation or trapping of particles and cells, the control of their trajectories or their encapsulation in droplets [23,24,25]. By this approach, particles subjected to an ultrasonic field, generated via bulk acoustic waves (BAW) or surface acoustic waves (SAW), are scattered or impinged by field waves, allowing the creation of an acoustic radiation force able to move particles towards the surrounding medium. In previous works, BAW devices were employed to separate blood components throughout a continuous and a biocompatible approach [26,27,28,29,30,31,32], thus validating a label-free approach [17] for biomarker detection.

This work investigated the performance of a novel bulk acoustic wave device, characterized by an innovative design, which could be employed to separate biomarkers possessing micrometric dimensions (i.e., circulating tumor cells, platelets, apoptotic bodies). Design and fabrication methods of the silicon-based microfluidic device are reported, as well as protocol steps involved in the development of a customized set-up to allow the formation of an acoustic standing wave field into the microfluidic channel. An evaluation of the critical particles’ diameter was carried out by investigating the focusing performance at sub-micron diameters. Moreover, mixed populations composed either of polystyrene micro- and nanoparticles or cells were tested at different experimental conditions (i.e., sample concentration, applied voltage value at the piezoelectric element and flow rate), thus giving a complete view of the potentiality of the device and the method, which is hard to find in the literature. Finally, the evaluation of the acoustic energy density allowed for a comparison between experimental and numerical calculation, confirming the possibility to predict the focusing performance at different concentrations.

The experimental data obtained from HL-60 cells demonstrated the capability to collect cells either at a high flow rate or when they are mixed with a population of micro and nanoparticles without affecting device focalization performances. Compared to the literature [30,31,32], focalization results performed with the presented acoustic wave device were obtained without the buffer flow confinement contribution. Thus, further improvements on this work can lead to the development of rapid and efficient diagnostic tools for micrometric biomarker separation in liquid biopsy.

## 2. Background Theory

Commonly, acoustic radiation force is divided into two components: primary and secondary radiation forces. Primary forces derive from the interaction between the incident wave and particles in the suspended medium, and secondary forces, in contrast to primary forces, refer to scattered wave interactions with other particles [21,33,34]. Since this work shows a bulk acoustic wave device able to separate cells and micro- and nanoparticles, a brief introduction on how it works is reported in the following.

BAW devices possess a basic configuration composed of a microfluidic channel with two parallel and opposing walls to perform acoustophoresis. Indeed, these elements are not only fluidic boundaries for the flow, but they also behave as reflectors (acoustic boundary) for waves propagating in the fluid. Thus, when bulk waves reflect at the fluid/structure interface, a superposition of an incident and a reflected propagating wave in the microfluidic channel results in an ultrasonic standing wave field [35,36,37]. Therefore, suspended particles move to the field pressure node or anti-node depending on the primary acoustic radiation force and mechanical characteristics (i.e., size, density, compressibility) of particles and surrounding fluid. This force acting on compressible spherical objects in a standing wave field, referring from the literature [38,39,40,41,42], can be defined as:

(1) $$F_{R}=-\left(\frac{\pi p_{0}^{2}V_{p\beta_{p}}}{2\lambda }\right)\phi \left(\beta ,\rho \right)sin\left(\frac{4\pi x}{\lambda }\right),$$

(2) $$\phi \left(\beta ,\rho \right)=\frac{5\rho_{p}-\rho_{m}}{2\rho_{p}+\rho_{m}}-\frac{\beta_{p}}{\beta_{m}}.$$

where $p_{0}$ is the acoustic pressure derived from the standing waves, $V_{p}$, $\beta_{p}$ and $\rho_{p}$ are the volume, the compressibility and the density of particles, $\beta_{m}$ and $\rho_{m}$ are the compressibility and density associated with the surrounded fluid and, finally, *ϕ*, *λ* and x are the acoustic contrast factor, the wavelength of the acoustic wave and the distance from a pressure node, respectively. Thus, acoustic primary force acts on particles possessing a diameter lower than the acoustic wavelength and moves them toward the node or the anti-node of the standing wave field, depending if the acoustic contrast factor value is positive or negative, respectively.

In microfluidic systems, dominated by a low Reynolds number, one has to also take into account another type of force acting on particles suspended in an aqueous solution. This force, given by the viscous attenuation of the suspended medium, corresponds to the Stokes drag force, and it is expressed as [43]:

(3) $$F_{D}=-6\pi \eta_{m}a_{0}v_{p},$$

where $\eta_{m}$ corresponds to the fluid viscosity, while $a_{0}$ and $v_{p}$ refer to the size and speed of the particle, respectively.

Thus, since the primary radiation force is proportional to the volume of particles while the drag force is proportional to its radius, as particle size decreases, Stokes force prevails over the acoustic force, becoming the predominant phenomenon acting on particles in the systems. Therefore, equalizing the primary acoustic radiation force and the Stokes force, it is possible to define the critical particle size diameter ($2a_{0}$), below which particles cannot be collected at the pressure node of the standing wave field [43,44,45,46]:

(4) $$2a_{0}=\sqrt{\frac{3\eta_{m}}{\phi \rho_{m}\pi f}}.$$

where f refers to the frequency associated with the acoustic wavelength.

### 2.1. Transverse Particle Path

Solving the differential equation derived from balancing the previous forces and by separating y and t components, one can also derive the analytical expression for the transverse particle path [47,48,49]:

(5) $$y\left(t\right)=\frac{1}{k}arctan\left\{tan\left[ky\left(0\right)\right]exp\left[\frac{4\phi }{9\eta_{m}}{\left(kr\right)}^{2}E_{ac}t\right]\right\},$$

where y(t) is the transverse position at time t, $k=\frac{2\pi }{\lambda }$ is the wave number along the y-component and $E_{ac}$ corresponds to the acoustic energy density of the system.

Inverting the above expression allows us to determine the acoustic energy density needed to move particles from any initial position y(0) (i.e., where the particle is located before the actuation of the acoustic field) to the pressure node of the system y(t):

(6) $$E_{ac}=\frac{9\eta_{m}}{4\phi {\left(kr\right)}^{2}t}ln\left[\frac{tan\left[ky\left(t\right)\right]}{tan\left[ky\left(0\right)\right]}\right],$$

By inserting this value in Equation (1), it is possible to define the pressure amplitude into the microfluidic channel:

(7) $$p_{a}=2\sqrt{E_{ac}\rho_{m}c_{m}^{2}}.$$

where $c_{m}$ refers to the surrounded medium sound velocity where particles are suspended.

### 2.2. Transversal Resonator

The design of the BAW device corresponds to a transversal resonator [50]. The microfluidic device is excited by a characteristic frequency that leads to the formation of an ultrasonic standing wave across the microchannel width perpendicular to the direction of actuation. This frequency matches the half wavelength criterion with respect to the channel width. In detail, this BAW device exploited first resonance modes characterized by a pressure node along the center of the microfluidic channel and pressure anti-nodes along its side walls. In this way, particles and cells characterized by a positive acoustic contrast factor [51] moved to the center of the microfluidic channel.

Then, according to literature [36,52,53,54], the width of the microfluidic channel $w_{ch}$ is designed as:

(8) $$w_{ch}=\frac{\lambda_{med}}{2}.$$

where $\lambda_{med}$ is the wavelength of the acoustic wave in a characteristic suspended medium. It derives from the ratio between the sound speed of the suspended medium $c_{med}$ and the resonant frequency f of the device.

## 3. Materials and Methods

### 3.1. Design

Considering a piezoelectric element whose working frequency is in the 4 MHz range and water as the suspended medium [44], the device dimensions are 14.50 mm in width, 76.54 mm in length and with a height of 1.05 mm. It consists of two symmetric inlets and outlets, with 500 µm of hole diameter, and a straight channel 40 mm long having a rectangular cross section 190 µm wide and 95 µm deep. In correspondence to the straight channel, the device wall width presents narrower dimensions to allow the formation of the standing wave field across the microfluidic channel [47]. This value, equal to an even number of the acoustic wavelength, is 530 µm.

The analytical value of the minimum particle diameter that could be collected at the node of the microfluidic channel is 1.6 µm. This is given by solving Equation (4).

### 3.2. Fabrication and Device Assembly

This BAW resonator relies on reflections between channel walls, so it needs high characteristic acoustic impedance materials [53]. Due to this, the bottom substrate of the BAW device is manufactured in silicon by using a standard microfabrication approach, since a precise channel structure with vertical walls is required.

**Figure 1:**
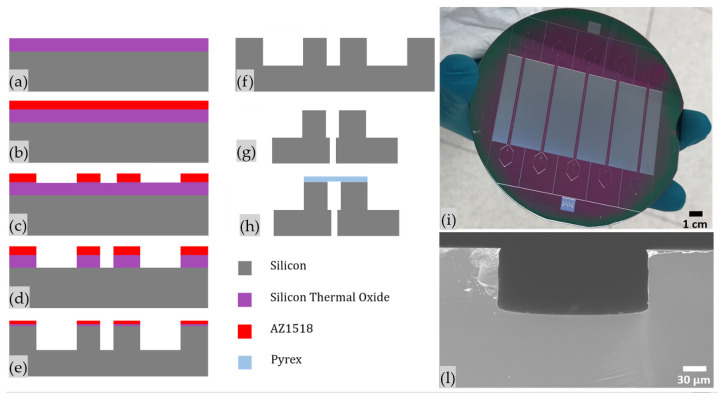
Chip Process Flow: (**a**) starting substrate cleaning, (**b**) photoresist spin coating, (**c**) photoresist exposure and development, (**d**) buffer oxide etching, (**e**) silicon deep reactive ion etching, (**f**) buffer oxide etching, (**g**) inlet/outlet laser drilling and chip dicing, (**h**) anodic bonding, (**i**) BAW devices after DRIE process and (**l**) Field Emission Scanning Electron Microscope (FESEM) cross section image of the separation channel.

A 4-inch n-type silicon wafer with (100) orientation and 0.35 mm thickness finished with 1 µm of thermal SiO_2_ is used. First, the adhesion promoter (Ti Prime, Microchemicals GmbH, Ulm, Germany) is coated on the silicon wafer by using a spin coater (Spinner 150 Wafer Spinner, SPS, Putten, The Netherlands) by setting 5 s at 500 rpm and 30 s at 4000 rpm to guarantee a perfect adhesion between the silicon surface and the photoresist used as a mask. This is followed by a soft bake on a hot plate at 120 °C for 2 min. Next, the wafer is spin coated by a positive photoresist AZ1518 (Microchemicals GmbH, Ulm, Germany) by setting 5 s at 500 rpm and 30 s at 4000 rpm to define a mask with an average thickness of 1.41 µm. The photoresist is exposed for 10 s through standard UV photolithography by means of a double side mask aligner (Neutronix Quintel NXQ 4006, Morgan Hill, CA, USA) used in contact mode, ensuring the correct alignment between the photoresist and the desired mask pattern. Next, the photoresist is developed using a solution of 1:4 AZ400K developer (Microchemicals GmbH, Ulm, Germany) in deionized water for 40 s, then rinsed twice with deionized water and dried under a nitrogen flux. After that, Buffer Oxide Etching (BOE) is performed for 15 min to remove the thermal oxide in the unwanted area and the microchannel etching is completed by Deep Reactive Ion Etching (DRIE) (Oxford Plasmalab 100 System, Oxford Instruments, Abingdon-on-Thames, UK). A Bosch^®^ process is performed to obtain an about 90 µm deep microchannel and hollows along it with highly vertical sidewalls, using the following parameters: 1500 W of ICP power, 10 W of RF power, 50 sccm of C_4_F_8_ for the passivation step extent of 4 s and 150 sccm of SF_6_ for the etch step extent of 7 s, imposing 14 sccm of He backside cooling to maintain 18 °C on the wafer in both steps. The etched silicon wafer is then submerged into a solution of sulfuric acid and hydrogen peroxide in a 3:1 ratio (*v*/*v*) for 5 min to remove the residual AZ1518 mask layer, rinsed 3 times in water and dried with a nitrogen flux. A further BOE process is needed to remove the residual thermal oxide after the DRIE step. Device and inlet/outlet dicing and drilling are achieved by laser etching (50 W G4 Pulsed Fiber Laser, Infra 1064 nm) and finally devices are sealed with 500-µm-thick slices of borosilicate glass (Corning 7740) by anodic bonding. PDMS interconnections are fixed on the bottom of the microfluidic device in correspondence to inlets and outlet ports to ensure a stable connection with polyurethane (PU) tubes (SMC OD = 2.0 mm, ID = 1.2 mm) and the device (Figure 1).

Ultrasonic standing waves are generated by a piezoelectric plate (CuNi 20 × 20 × 0.5 mm^3^ with screen printed Ag electrodes from Physik Instrumente, Karlsruhe, Germany) with a nominal resonance frequency of 4 MHz. This element, located on the back side of the microfluidic channel, ensures a continuous flow of separated particles. Such an arrangement between the microfluidic device and the piezoelectric plate allows a large contact surface area, empowering a good coupling of the acoustic energy into the chip [52]. Finally, a thin layer of ultrasound gel (Shockwave Gel from ELvation Medical GmbH, Kieselbronn, Germany) is employed between the transducer (piezoelectric plate) and the rear side of the microfluidic channel to improve the acoustic coupling. Indeed, the application of an ultrasound gel not only minimizes the acoustic losses, but also allows the use of the same transducer several times [54].

### 3.3. Experiment Setup and Samples

**Figure 2:**
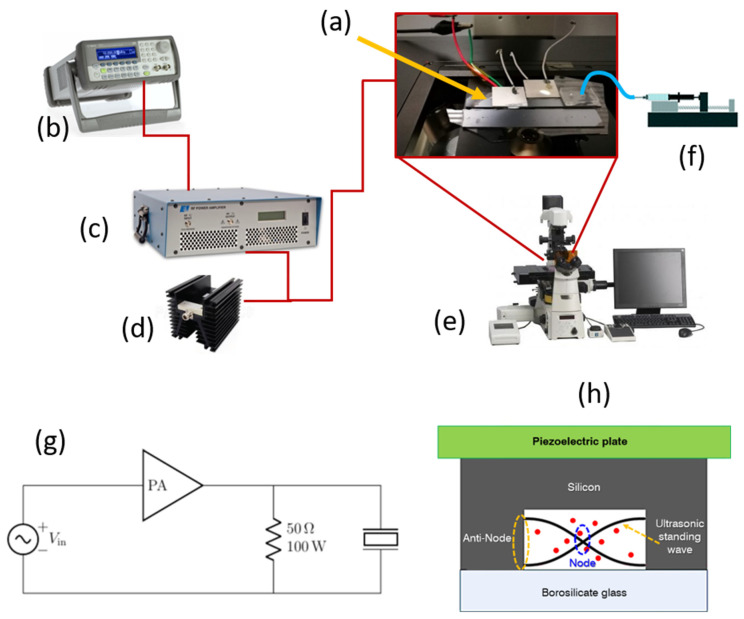
Experimental setup: (**a**) BAW device and resonating plate in which: the light blue line indicates the MP solution and the red one the electrical connections to actuate the piezoelectric elements (white), (**b**) waveform generator, (**c**) RF power amplifier, (**d**) dummy load, (**e**) optical fluorescence microscope, (**f**) syringe pump, (**g**) electrical diagram of the experimental setup used to generate the US standing wave into the microfluidic channel, (**h**) cross section sketch of the BAW device operating principle.

A customized experimental setup, composed of different elements, is assembled and illustrated in Figure 2. Tests presented in this work are performed by exploiting only one inlet access. A syringe pumping system (Harvard Apparatus 11 Plus, Harvard Apparatus, Holliston, USA) is used to inject analytes through the device. The piezoelectric element is actuated by applying a harmonically oscillating peak-to-peak voltage (Vpp) generated by a waveform generator (Agilent 33220A). In particular, the voltage is amplified by 50 dB by a radio frequency (RF) power amplifier (E&I Ltd., Rochester, USA, 2100L 10 KHz–12MHz, 100 W) connected to a dummy load terminator (50 Ω, 100 W). During experiments, the microfluidic channel is actuated at its resonance frequency of 4.623 MHz (see Supplementary Materials Figures S1 and S2). Moreover, the microfluidic channel is monitored and time lapses are acquired through a sCMOS camera (Hamamatsu, Hamamatsu City, Japan) of a fluorescence microscope (Nikon Eclipse Ti-E Inverted, Nikon, Minato, Japan) with a 4× objective lens.

Performances of the BAW device are evaluated by employing different types of particles. Four-micrometer fluorescent sulfated polystyrene microparticles (from now on called 4MPs) (FluoSpheres™, Thermo Fisher Scientific, Waltham, MA, USA) diluted in water with 0.01% Tween20 (Sigma-Aldrich, St. Louis, MO, USA) are used with concentrations equal to 5.68 × 10^6^ particles/mL, 1.14 × 10^6^ particles/mL and 5.68 × 10^5^ particles/mL. One-micrometer fluorescent sulfated polystyrene microparticles (from now on called 1MPs) (FluoSpheres™, Thermo Fisher Scientific, Waltham, MA, USA) diluted in water with 0.01% Tween20 are employed with a concentration of 1.00 × 10^6^ particles/mL. Finally, a mixed population of micro and nanoparticles characterized by 4MPs and 500-nm fluorescent carboxylated polystyrene nanoparticles (from now on called NPs) (from Magsphere Inc., Pasadena, CA, USA) diluted in water with 0.01% Tween20 is used. In this latter case, 4MPs’ batch concentration is equal to 5.68 × 10^5^ particles/mL, while NPs’ batch concentration corresponds to 3.64 × 10^5^ particles/mL.

Cells employed for proof-of-concept experiments are HL-60 cells (ATCC^®^ CCL-240^TM^, Manassas, VA, USA) obtained from an acute promyelocytic leukemia patient. Cells are grown in suspension and maintained in Iscove’s Modified Dulbecco’s Medium (Sigma-Aldrich, St. Louis, MO, USA) supplemented with 20% heat-inactivated FBS (Sigma-Aldrich, St. Louis, USA), 1% L-Glutamine (Sigma-Aldrich), 100 units/mL penicillin and 100 µg/mL streptomycin (Sigma-Aldrich, St. Louis, MO, USA) in 25–75 cm^2^ non-treated cell culture flasks (Corning Inc., Corning, NY, USA) in a cell incubator at 37 °C in a humified atmosphere containing 5% CO_2_.

The following protocol is employed to label cells. First, cells are counted and a certain number of cells are pelleted by centrifugation at 130× *g* for 5 min and resuspended in 500 µL of phosphate-buffered saline (PBS, Sigma-Aldrich, St. Louis, MO, USA) to reach one of the desired densities between 5.00 × 10^6^ cells/mL, 1.00 × 10^6^ cells/mL and 5.00 × 10^5^ cells/mL. Then, plasma membranes of cells are labeled with WGA (Wheat Germ Agglutinin) conjugated with Alexa Fluor 647 dye (Thermo Fisher, Waltham, MA, USA). In detail, 2.5 µL of WGA (1 mg/mL, *w*/*v*) is added to cells in PBS solution to reach a concentration of 5 µg/mL, as recommended by the manufacturer, and they are placed in an orbital shaker at 37 °C set at 50 rpm for 10 min in the dark. Later, as the washing step to remove the unlabeled dye, cells are centrifuged at 130× *g* for 5 min and resuspended in 500 µL of PBS.

Before each experiment and between each run, the device is rinsed with bi-distilled water (MilliQ, from now water) at a flow rate of 30 µL/min for 10 min. Regarding proof-of-concept experiments, since cells are dispersed into BS solution at 7.4 pH, the last washing steps either before the experiment or between each run are performed with PBS.

### 3.4. Focusing Characterization Methods

When the device is stimulated at its resonance frequency, various conditions are taken into account to evaluate its focalization performance at the node of the acoustic standing wave either with particles or cells. In detail, experiments are performed with a constant resonant frequency and by varying one of the following parameters for each test: sample concentration or type, flow rate and applied voltage at the piezoelectric element.

Then, the focusing capability of the BAW device is determined by analyzing samples collected at the outlets by two different approaches. The first approach analyzes samples through the ratio between the absorbance of elements collected at the central outlet solution (node) and the total absorbance of elements at the two outlets. Absorbance values are quantified by a UV–Vis characterization, as already explained in our previous paper, where calibration curves for particle absorbance characterizations are also reported [55]. Concerning cells and cells mixed with particles, calibration curves are performed by setting 650 nm as the impinging wavelength to evaluate cell absorbance values (see Supplementary Materials Figures S3 and S4).

The other way to evaluate the performance of the chip to collect particles at the node is determined by analyzing images acquired during a time lapse by exploiting the co-localization program (Nis-Element from Nikon, Minato, Japan) of the fluorescence microscope. By this, sample analysis is available when thresholds between background fluorescence intensities and samples sizes are defined. Then, the focusing is expressed as the ratio between the counted elements localized at the center of the microfluidic channel (node) and the counted elements in the microfluidic channel. Time lapses are acquired in a defined focal plane of the 4× objective lens (1.63 µm/pixel). Regarding 4MPs, they are implemented for 50 s: images are captured every 300 ms with an exposure time of 9.8 ms in the green channel (FITC-A filter). HL-60 cell time lapses are implemented for 30 s; thus, images are acquired every 50 ms with the same exposure time in the near infrared (NIR) channel (Cy5-4040C filter). Finally, for the mixed population of 1MPs and HL-60 cells, time lapses are acquired every 30 ms setting an exposure time of 9.8 ms in the green channel for the 1MPs and an exposure time of 20 ms in the NIR channel for the HL-60 cells.

During tests performed with HL-60 cells, a third approach is used to evaluate the performance of the BAW device. This is conducted by manually counting cells collected at the outlets through a Bürker counting chamber. Briefly, 10 µL of the cell suspension is placed in the Bürker chamber and cells are manually counted as cell density, intended as number of cells/mL, and thus the total number of cells collected at the two outputs and the percentage of focalization are assessed [56].

To end, experiments with the same conditions are repeated at least three times, thus error bars are reported according to the acquired data over the repetitions.

### 3.5. Experimental Determination of the Acoustic Energy Density and Local Pressure Amplitude

The experimental value of the acoustic energy density is determined by analyzing the transient acoustophoretic focusing of particles [43,47]. Time lapses of 4MPs concentrated at 5.68 × 10^6^ particles/mL are implemented for 2 minutes while the ultrasounds are turned off (Vpp = 0 V) and on (Vpp = 52.92 V). Images are captured every 500 ms with an exposure time of 9.8 ms in the green channel. Then, these videos are elaborated by an open source video analysis software called Tracker 2.6 (from Open Source Physics by D. Brown), which allows the extraction of the analytical expression of the transverse path y(t) of each particle captured in the video frame to frame. Finally, the estimation of the acoustic energy density value is given by mediating the list of (*t*, *y*)-coordinates of 20 particle paths for the defined time needed to move particles at the nodes and solving Equation (6). Knowing the acoustic energy density value, the estimation of the pressure amplitude in the BAW device is obtained by solving Equation (7).

### 3.6. Numerical Model

A numerical 3D model is implemented to perform tests about the particle focusing. To avoid the high computational demand required by a full 3D numerical model, we used the limiting velocity finite element method [57]. In this efficient approach, the acoustic streaming is predicted only outside the viscous boundary layer and thus the mesh resolution inside has to not be high, leading to a very coarse mesh. The first-order acoustic fields are implemented using the built-in COMSOL’s interface “Pressure Acoustics”, which solves the following harmonic equation:

(9) $$\nabla^{2}p=-\frac{\omega^{2}}{c^{2}}p,$$

where p is the pressure field, c is the speed of sound in water and ω is the angular frequency. The actuation of the walls is introduced as a boundary condition at the side walls through a normal harmonic displacement.

Through the first-order fields, is it possible to compute the limiting velocities, using the following equations [57]:

(10) $$u_{L}=-\frac{1}{4\omega }Re\;\left\{q_{x}+u_{1}^{*}\left[\left(2+i\right)\left(\frac{du_{1}}{dx}+\frac{dv_{1}}{dy}+\frac{dw_{1}}{dz}\right)-\left(2+3i\right)\frac{dw_{1}}{dz}\;\right]\right\},$$

(11) $$v_{L}=-\frac{1}{4\omega }Re\;\left\{q_{y}+u_{1}^{*}\left[\left(2+i\right)\left(\frac{du_{1}}{dx}+\frac{dv_{1}}{dy}+\frac{dw_{1}}{dz}\right)-\left(2+3i\right)\frac{dw_{1}}{dz}\right]\right\},$$

(12) $$q_{x}=u_{1}\frac{du_{1}^{*}}{dx}+v_{1}\frac{du_{1}^{*}}{dy},$$

(13) $$q_{y}=u_{1}\frac{dv_{1}^{*}}{dx}+v_{1}\frac{dv_{1}^{*}}{dy},$$

where $u_{L}$ and $v_{L}$ are the two components of the limiting velocities, while $u_{1},v_{1}$ and $w_{1}$ are the components of the three-dimensional first-order velocity field.

The second-order fields are simulated using COMSOL’s physics “Creeping Flow”. Considering a Stokes flow, the equations take the form:

(14) $$\nabla p_{2}=\mu \nabla^{2}v_{2},$$

(15) $$\nabla \cdot v_{2}=0.$$

## 4. Results and Discussion

### 4.1. Particle Focusing Analysis

**Figure 3:**
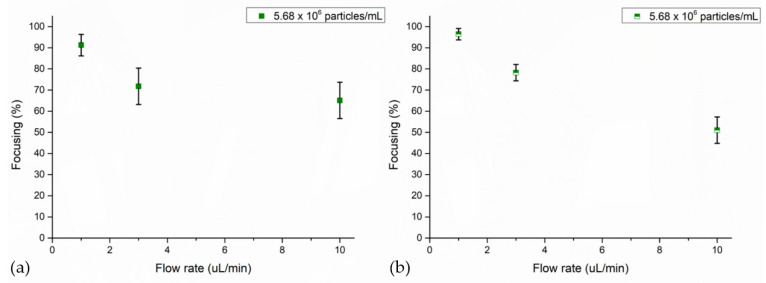
Focusing performance of the BAW device with 4MPs concentrated at 5.68 × 10^6^ particles/mL injected at 1 μL/min, 3 μL/min and 10 μL/min: (**a**) UV–Vis characterization and (**b**) image analysis. Experiments were performed by applying 50.59 Vpp at the transducer while the device was actuated at its resonant frequency of 4.623 MHz.

Referring to the literature [47,52], a first set of experiments was performed with 4MPs concentrated at 5.68 × 10^6^ particles/mL dispersed in water and injected at different flow rates. Tests were carried out by applying 50.59 Vpp to the transducer by actuating the microfluidic channel at its resonance frequency. These focalization tests were performed to investigate one by one the optimal setting values to impose to reach the maximum achievable collection of particles at the pressure node of the BAW device. Focusing values at low flow rates lead to an increased collection of particles at the pressure node of the standing acoustic wave field, as shown throughout a UV–Vis analysis. Indeed, at 1 μL/min it was 91%, while for 3 μL/min and 10 μL/min it corresponded to 72% and 65%, respectively (Figure 3a). A further proof of this focusing trend is also detected by the image analysis, where for each experiment, and thus each time lapse, values are mediated, evaluating 20 equidistant images of a time lapse. In this case, the calculated focusing percentage values are 96% at a flow rate of 1 μL/min, 78% for a flow rate equal to 3 μL/min and 51% when 4MPs moved at 10 μL/min (Figure 3b). Low flow rates led suspended particles to be subjected to the ultrasound field for a longer time period while traveling through the microfluidic channel, allowing more particles to reach the pressure node [21].

**Figure 4:**
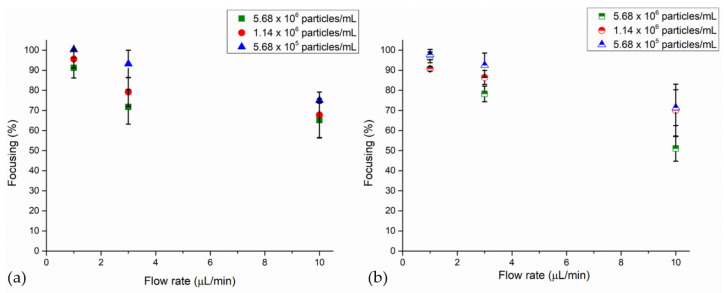
Focusing performance of the BAW device as a function of flow rates and 4MP concentrations: (**a**) UV–Vis characterization and (**b**) image analysis. Experiments were performed with 4MPs concentrated at 5.68 × 10^6^ particles/mL, 1.14 × 10^6^ particles/mL and 5.68 × 10^5^ particles/mL. During the experiments, 4MPs were injected at 1 μL/min, 3 μL/min and 10 μL/min, while 50.59 Vpp was applied at the transducer when the device was actuated at its resonant frequency of 4.623 MHz.

A similar trend of particles collected at the node of the BAW device is noticed by exploiting two other 4MP concentrations when different flow rates are investigated. Thus, leaving other settings constant, tests accomplished with different particle concentrations showed that lower concentrations promoted a higher collection of particles at the node of the microfluidic channel (Figure 4). Indeed, 100% and 96% of focusing percentage values are characterized via the UV–Vis method when 4MPs concentrated at 1.14 × 10^6^ particles/mL and 5.68 × 10^5^ particles/mL are injected at 1 μL/min. At 3 μL/min, these values, performed throughout the UV analysis, were 80% and 93% for 4MPs concentrated at 1.14 × 10^6^ particles/mL and 5.68 × 10^5^ particles/mL, respectively. A confirmation of these results is given by the image analysis, where a maximum discrepancy of 6% is detected between average values of the two characterization methods. At last, the percentages of particles collected at the node of the BAW device at 10 μL/min were 68% and 75% for a concentration of 1.14 × 10^6^ particles/mL and 5.68 × 10^5^ particles/mL through the UV–Vis analysis. Throughout image analysis characterization, these values were 70% and 71%. Thus, a negative effect on the device’s focusing performance is correlated to higher concentrations when particles collected at the pressure node saturated it, causing an increase in inter-particle forces and thus requiring a stronger acoustic force [37,58,59].

**Figure 5:**
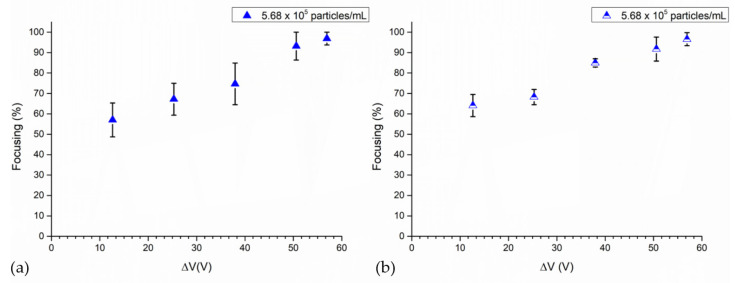
Focusing performance of the BAW device as a function of applied voltages at the transducer: (**a**) UV–Vis characterization and (**b**) image analysis. During the experiments, 4MPs concentrated at 5.68 × 10^5^ particles/mL were injected at 3 μL/min into the device actuated at its resonant frequency of 4.623 MHz, while 56.92 Vpp, 50.59 Vpp, 37.95 Vpp, 25.29 Vpp and 12.65 Vpp were applied at the transducer.

A second set of tests observed changes in the focusing performance of the BAW device when the following voltages were applied to the piezoelectric element: 56.92 Vpp, 50.59 Vpp, 37.95 Vpp, 25.29 Vpp and 12.65 Vpp. During these experiments, 4MPs, concentrated at 5.68 × 10^5^ particles/mL, were injected at 3 μL/min when the microchannel was actuated at its fundamental resonance frequency. Higher voltages are associated with higher focusing values, as reported either by UV–Vis characterization or image analysis (Figure 5). In detail, when 12.65 Vpp was applied to the piezoelectric element, the focalization was 57% for the UV–Vis analysis and 64% via the image analysis, while when 56.92 Vpp was set to the transducer 97% of particles converged to the pressure node of the microfluidic channel, as shown by both characterization techniques. At 25.29 Vpp and 37.95 Vpp, focusing values were 67% and 75% for the UV–Vis characterization, while through image analysis they were 68% and 85%, respectively.

Focalization values performed throughout UV–Vis characterization and image analysis are useful approaches able to describe the focalization performance of the BAW device. Indeed, results performed by these techniques showed a maximum discrepancy of 10%.

**Figure 6:**
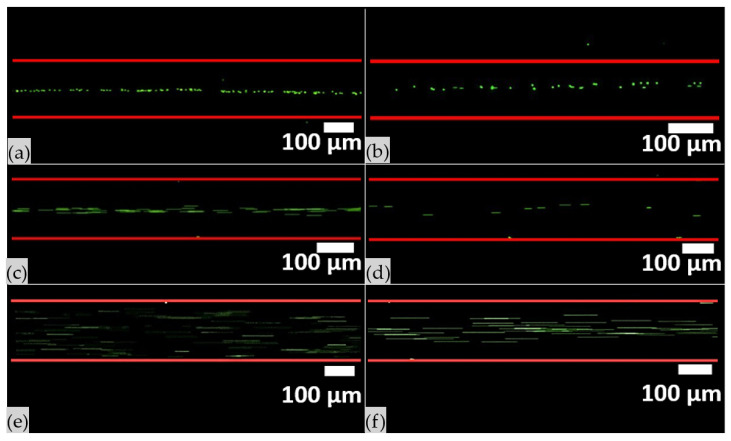
Frame of time lapse acquired during the acoustophoretic test, when 4MPs were employed at different concentrations and flow rates: (**a**) 4MPs concentrated at 5.68 × 10^6^ particles/mL were injected at 1 µL/min, (**b**) 4MPs concentrated at 1.41 × 10^6^ particles/mL were injected at 1 µL/min, (**c**) 4MPs concentrated at 5.68 × 10^6^ particles/mL were injected at 3 µL/min, (**d**) 4MPs concentrated at 1.41 × 10^6^ particles/mL were injected at 3 µL/min, (**e**) 4MPs concentrated at 5.68 × 10^6^ particles/mL were injected at 10 µL/min and (**f**) 4MPs concentrated at 1.41 × 10^6^ particles/mL were injected at 10 µL/min. Experiments were performed by applying 50.59 Vpp at the transducer, while the device was actuated at its resonant frequency of 4.623 MHz. Images acquired with 4× objective lens with an exposure time of 9.8 ms.

Finally, observing the time-lapse images, as long as particle concentration decreased, the acoustic force increased, inducing particles to accumulate in a narrower band along the pressure node (Figure 6). An analogous effect was also observed when the voltage applied to the piezoelectric element increased; indeed, a narrower band along the pressure node is detected at higher voltages.

**Figure 7:**
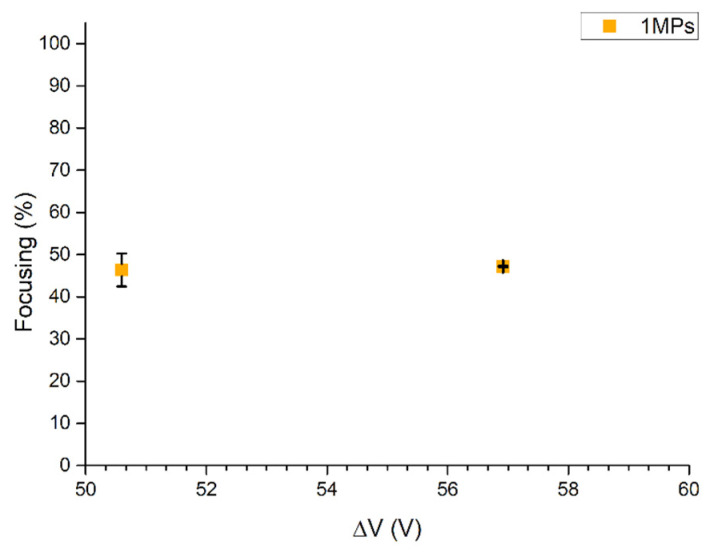
UV–Vis characterization of focusing performance of the BAW device with 1MPs concentrated at 1.00 × 10^6^ particles/mL injected at 1 μL/min when the device was actuated at its resonance frequency of 4.623 MHz. Experiments were performed by applying 50.59 Vpp and 56.92 Vpp at the transducer.

Further focalization tests were carried out to experimentally check the critical particle diameter 2$a_{0}$ derived from Equation (4). Thus, for these experiments, a dispersion of 1MPs concentrated at 1.00 × 10^6^ particles/mL was injected at 1 μL/min into the BAW device actuated at its resonance frequency when different voltages were applied to the piezoelectric element. Voltage values of 50.59 Vpp and 56.92 Vpp were selected according to the maximum collection of particles at the node of the BAW device from previous experiments’ results. Both cases showed a low focalization value in accordance with the analytical result of 1.6 µm. Indeed, it corresponded to 46% and 47% when 50.59 Vpp and 56.92 Vpp were applied to the transducer, respectively (Figure 7). Therefore, this design cannot be employed to focalize particles characterized by sizes equal to or below 1.6 μm at the pressure node of the device.

**Figure 8:**
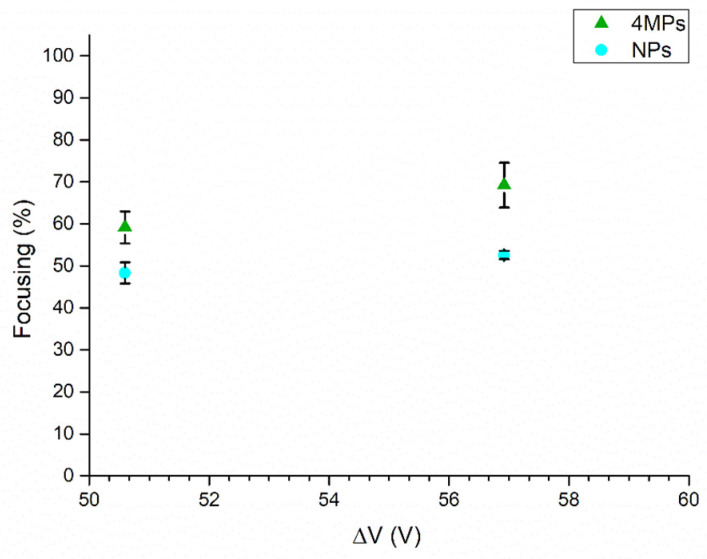
UV–Vis characterization of focusing performance of the BAW device when a mixed population of 4MPs concentrated at 2.84 × 10^5^ particles/mL and NPs concentrated at 1.82 × 10^5^ particles/mL was injected at 1 μL/min into the device, which was actuated at its resonance frequency of 4.623 MHz. Experiments were performed by applying 50.59 Vpp and 56.92 Vpp at the transducer.

A further proof regarding the experimental evaluation of the critical particle diameter 2$a_{0}$ was determined by analyzing a mixed population of 4MPs and NPs concentrated at 2.84 × 10^5^ particles/mL and 1.82 × 10^5^ particles/mL, respectively. Then, the mixed population of particles was fluxed at a flow rate of 1 μL/min into the chip, while it was actuated at its resonance frequency and when 50.59 Vpp or 56.92 Vpp voltages were applied to the piezoelectric element. The results showed focalization values of 59% at 50.59 Vpp and 69% at 56.92 Vpp for 4MPs, while for the same applied voltages NPs’ focusing percentage values were 48% and 52% (Figure 8). Thus, even if NPs’ focusing performance appeared to be higher compared to 1MPs’ focusing performance, this is due to the fact that NPs mixed with 4MPs and interacted with them, and so they were affected by the presence of microparticles and vice versa (i.e., surface electrostatic interactions, scatterings, second-order radiation forces) [58]. Indeed, evaluating the focusing value only for the 4MP population, concentrated at 5.68 × 10^5^ particles/mL and injected at 1 μL/min when 50.59 Vpp was applied at the transducer, it was 97%, a higher value related to 59% obtained with the mixed population.

### 4.2. Experimental Determination of the Acoustic Energy Density and Local Pressure Amplitude

**Figure 9:**
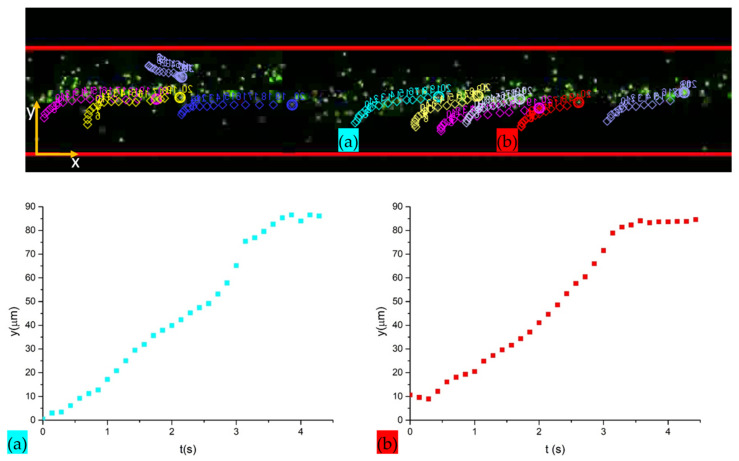
Tracking of bead paths with the Tracker 2.6 software and measurement of the transverse path *y*(*t*) from channel walls to the pressure node of two different beads (**a**) and (**b**). Measurements were performed in a specific focal plane of the 4× objective lens of the fluorescence microscope.

As mentioned before, the estimation of the acoustic energy density derives from the analytical expression of the transverse path *y*(*t*). Figure 9 displays different transverse paths *y*(*t*) of particles highlighted by blank circles of different colors inside the microfluidic channel in a time lapse extracted from the Tracker 2.6 video analysis tool. By mediating the list of (*t*, *y*)-coordinates of particle paths for the defined time needed to move particles from channel walls to the node, and inserting these values to Equation (6), an acoustic energy density of $7.25\pm 1.61\;\frac{J}{m^{3}}$ is obtained. Then, knowing the value of the acoustic energy density, a pressure amplitude of $p_{a}\approx 0.252\;MPa$ is estimated inside the chip.

### 4.3. Comparison between Experimental and Simulated Particle Focusing

**Figure 10:**
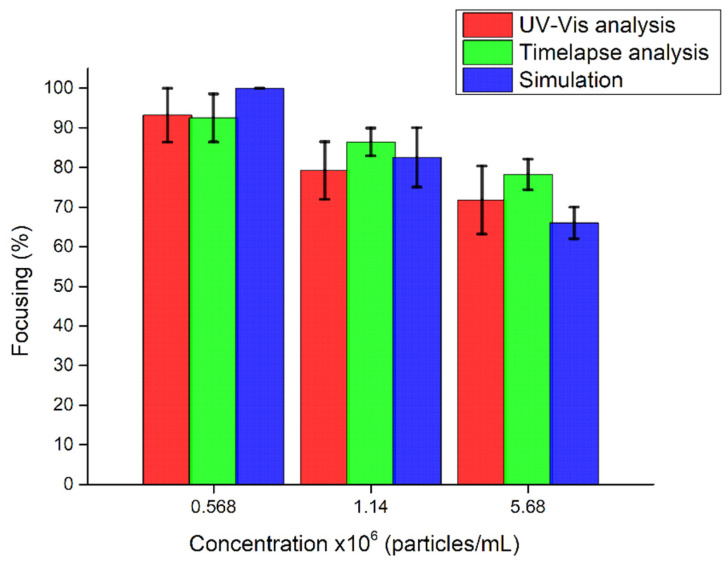
Comparison of the focusing performance of the BAW device obtained through simulations and experiments when 4MPs at different concentrations (5.68 × 10^6^ particles/mL, 1.14 × 10^6^ particles/mL and 5.68 × 10^5^ particles/mL) are investigated. Experimental values are reported, characterized either by UV–Vis analysis or image analysis.

Knowing the acoustic energy density from the calculation in the previous section, we used it for performing numerical tests to validate some experimental conditions. The acoustic radiation force and the Stokes drag force experienced by polystyrene particles were defined by setting the acoustic energy density at 7 Pa and computing the velocity and pressure acoustic fields. For each concentration, a fixed number of particles was injected from the inlet every second (29, 57 and 284, respectively), and the particles collected in the central outlet (defined by a region with a width equal to 1/3 of $w_{ch}$) were recorded over time. A comparison between the results obtained experimentally and numerically is reported in Figure 10. The recorded data showed a good match with promising percentages of focusing. As can be seen, an increase in the concentration of particles leads to a lower efficiency in focusing for both experimental and numerical tests. This is unexpected behavior, since an increasing number of particles leads to a higher possibility of particle–particle forces and thus to aggregation. Considering these aggregates as particles with higher sizes, the acoustic radiation force has to be stronger and move the particles towards the pressure node very quickly. Despite this, in these tests the acoustic energy density did not have high values, and thus the particles probably could not experience a strong acoustic radiation force. Thus, they moved slowly to the pressure node and a fraction of the total number of the particles remained at the side of the channel. In spite of this effect, the focusing efficiency is quite high for a particle concentration of 0.568 × 10^6^ particles/mL, reaching values close to 100%.

### 4.4. Biological Test

Proof-of-concept experiments were performed firstly with HL-60 cells as a sample solution and then with a mixed population of cells and MPs or NPs.

**Figure 11:**
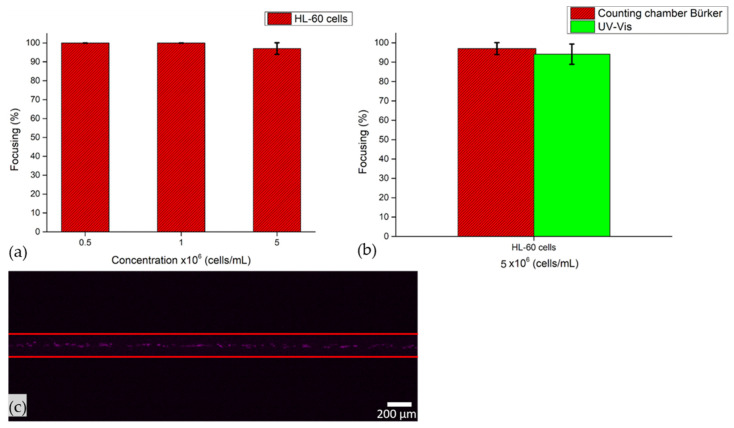
Focusing performance of the BAW device as a function of cell concentrations from Bürker counting chamber analysis. (**a**). Cells were injected at 3 µL/min when 50.59 Vpp was applied to the transducer, while the device was actuated at its resonance frequency of 4.623 MHz. (**b**) Focusing performance of the BAW device from Bürker counting chamber analysis and UV–Vis characterization. HL-60 cells concentrated at 5 × 10^6^ cells/mL were injected at 3 µL/min when 50.59 Vpp was applied to the transducer, while the device was actuated at its resonance frequency of 4.623 MHz. (**c**) Frame of time lapse acquired during the acoustophoretic test, when HL-60 cells concentrated at 5 × 10^6^ cells/mL were injected at 3 µL/min, 50.59 Vpp was applied to the transducer and the device was actuated at its resonance frequency of 4.623 MHz. Image acquired with 4× objective lens with an exposure time of 30 ms.

At first, HL-60 cells dispersed in a PBS solution at the concentrations of 5 × 10^5^ cells/mL, 1 × 10^6^ cells/mL and 5 × 10^6^ cells/mL were used to evaluate the focusing performance of the device with biological samples. Tests were carried out by applying 50.59 Vpp to the transducer, actuating the microfluidic channel at its resonant frequency and by injecting the solution of cells at a flow rate equal to 3 µL/min. From Bürker counting chamber analysis, focusing performance is verified at all concentrations. Indeed, the 100% of focusing was either at 5 × 10^5^ cells/mL or 1 × 10^6^ cells/mL, while at 5 × 10^6^ cells/mL the highest value of cell concentration was 97%, suggesting that at this flow rate value cells are easily collected at the pressure node of the device, even at higher concentrations. The latter result was also demonstrated through a UV–Vis characterization and image analysis. Indeed, 94% of cells are collected at the node of the device via UV–Vis characterization, while the time lapse showed cells in line at the node of the microfluidic channel when the acoustic field is applied (Figure 11).

This cell concentration was selected among the other ones either to compare this value with those obtained with micro- and nanoparticle tests or to collect a higher number of cells at the outlets after focalization tests, so as to better characterize them in the optics of developing this device as a Lab-On-Chip (LOC).

**Figure 12:**
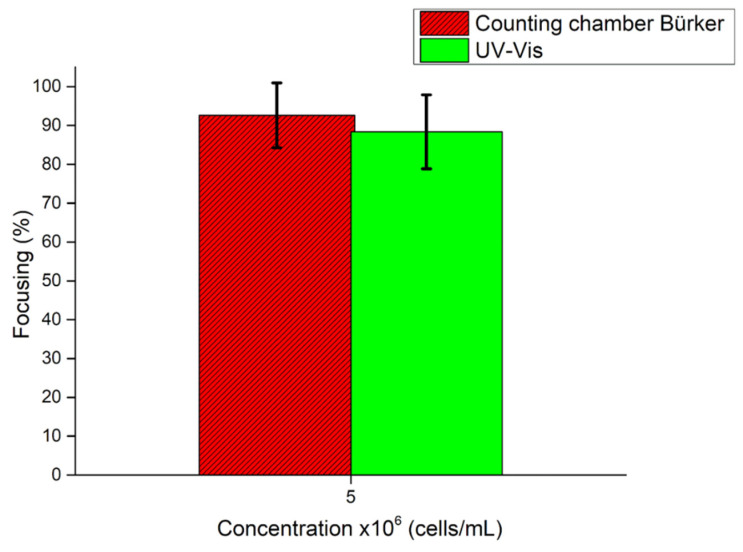
Focusing performance of the BAW device from Bürker counting chamber analysis and UV–Vis characterization. HL-60 cells concentrated at 5 × 10^6^ cells/mL were injected at 3 µL/min when 50.59 Vpp was applied to the transducer, while the device was actuated at its resonance frequency of 4.623 MHz.

Then, HL-60 cells concentrated at 5 × 10^6^ cells/mL were injected at an increased flow rate of 20 µL/min into the device to investigate the focalization performance. Experiments were performed, leaving the same parameter of applied voltage as previous tests. UV–Vis analysis and a Bürker counting chamber analysis reported focusing values of 92% and 88%, respectively. Thus, these demonstrate the device’s capability to collect an increased number of cells in a reduced time without affecting the focalization performance (Figure 12).

Further tests were performed with a mixed population composed of HL-60 cells and 1MPs at first and then with HL-60 cells and NPs, both of them in a 1:1 cells:M/NPs ratio. Tests were executed by applying 50.59 Vpp to the transducer, actuating the microfluidic channel at its resonance frequency and by injecting solutions at a flow rate equal to 3 µL/min. This last value is preferred to 20 µL/min since by this a higher collection of cells at the node of the device can be achieved. The mixed population composed of cells and 1MPs was characterized by HL-60 cells with a density equal to 2.5 × 10^6^ cells/mL and 1MPs concentrated at 2.5 × 10^5^ particles/mL. Instead, the other population was composed of the same density of cells mixed with NPs concentrated at 1.8 × 10^5^ particles/mL.

Concerning focalization values of HL-60 cells, they were 92% when they were mixed with 1MPs and 88% when they were mixed with NPs (see Supplementary Materials for Bürker analysis characterization, Figure S5). Comparing cell focalization results obtained when they were mixed and when there were only cells dispersed in PBS, a small reduction is observed as in the case of micro- and nanoparticles. Then, the majority of cells were focalized even in the presence of specimens of other sizes, ensuring the capability of this device as a promising tool for diagnostic purposes.

**Figure 13:**
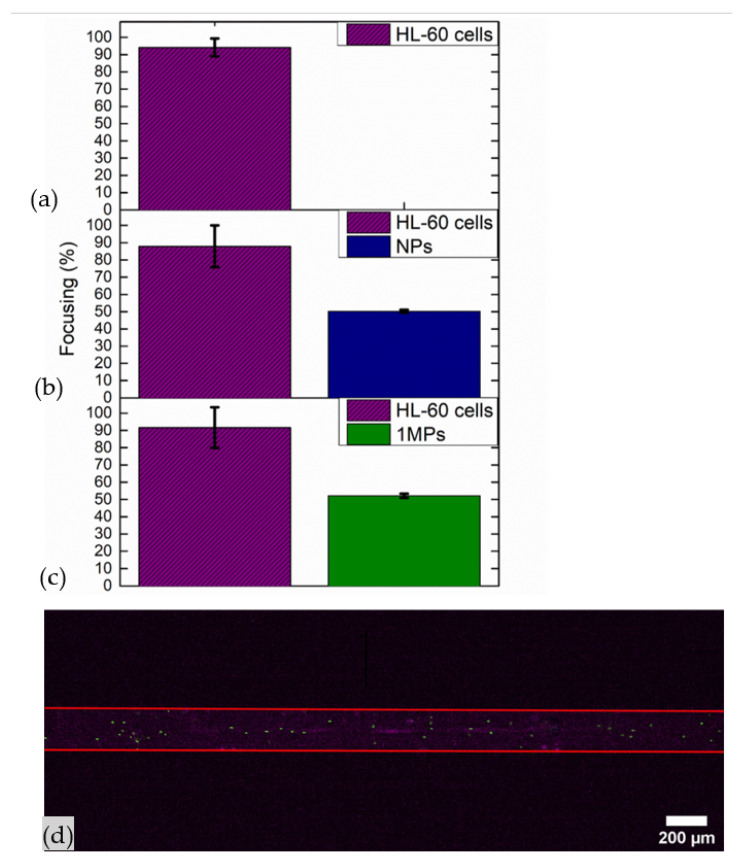
Focusing performance of the BAW device throughout a UV–Vis characterization: (**a**) HL-60 cells concentrated at 2.5 × 10^6^ cells/mL, (**b**) HL-60 cells concentrated at 2.5 × 10^6^ cells/mL mixed with 1MPs concentrated at 2.5 × 10^5^ particles/mL and (**c**) HL-60 cells concentrated at 2.5 × 10^6^ cells/mL mixed with NPs concentrated at 1.8 × 10^5^ particles/mL. Mixed populations were injected at 3 µL/min when 50.59 Vpp was applied to the transducer, while the device was actuated at its resonance frequency of 4.623 MHz. (**d**) Frame of time lapse acquired during the acoustophoretic test, when the mixed population of HL-60 cells concentrated at 2.5 × 10^6^ cells/mL and 1MPs 2.5 × 10^5^ particles/mL was injected at 3 µL/min, 50.59 Vpp was applied to the transducer and the device was actuated at its resonance frequency of 4.623 MHz. Image acquired with 4× objective lens with an exposure time of 20 ms for cells and 9.8 ms for 1MPs.

Additionally, in this case, as demonstrated previously with particles, the device was not able to collect 1MPs and NPs at the pressure node of the microfluidic channel. Indeed, the percentage of 1MPs and NPs focalized at the node was 52% and 50%, respectively (Figure 13).

## 5. Conclusions

This work investigated the separation potentiality of a silicon-based bulk acoustic wave resonator, characterized by a standard fabrication process as a label-free LOC. Focalization performances were evaluated by exploiting either polystyrene micro- and nanoparticles or adhesion cells as single or mixed populations. Samples collected at the outlets were characterized either via a UV–Vis method or throughout images analysis. The first class of experiments showed that 97% of microparticles were collected at the node of the microfluidic channel when a lower concentration, lower flow rate and higher applied voltage were imposed to the piezoelectric element. In addition, the microparticle focusing trend at different concentrations fitted well with the simulated ones obtained after having calculated the experimental acoustic energy.

As suggested from the analytical evaluation of the critical particle diameter, the ability to collect particles at the node of the microchannel worsens with particles of small sizes; indeed, the acoustic force is no longer able to focalize particles of sizes equal to or below 1.6 µm, as demonstrated either when samples were composed of a population of 1 µm particle sizes or when nanoparticles mixed with microparticles were exploited as samples.

Preliminary tests performed with cells highlighted an optimal separation performance even at a high flow rate. Indeed, at 20 µL/min, 92% of cells were focalized at the node of the BAW device. In addition, cell focalization performances were also demonstrated when they were used within micro- and nanoparticles. Indeed, average focalization values moved from 97%, when cells were alone, to 92% or 88% when they were mixed in samples with micro- and nanoparticles, respectively. Conversely to bulk acoustic wave devices presented in the literature, focalization results performed in this work were obtained without the buffer flow confinement contribution.

Finally, the presented device could be exploited in the future to develop an LOC for early diagnosis or some specific target therapies. Further work will be performed to optimize the separation between different kinds of cells or biomarkers owing to different mechanical properties such as shapes, sizes and densities. Thus, a buffer sheath fluid or a solution characterized by enhanced densities or viscosities with respect to the sample mechanical characteristics will be investigated.

## Data Availability

Data available on request due to privacy restrictions. The data presented in this study are available on request from the corresponding author. The data are not publicly available due to privacy reasons.

## Supplementary Materials

The following are available online at www.mdpi.com/article/10.3390/nano11102630/s1, Figure S1: Measurement of the displacement of the piezoelectric element performed throughout the laser Doppler vibrometer, Figure S2: Frame of time lapse acquired during the acoustophoretic test for the research of the exact resonance frequency of the BAW device. 4MPs concentrated at 5.68 × 10^6^ particles/mL are stable in water dispersion inside the microfluidic channel. (a) Ultrasound off and (b) ultrasound on. Images acquired with 4× objective lens with an exposure time of 9.8 ms. Figure S3: Calibration curve of HL-60 cells with regression equation $y=1.09\times 10^{-8}x$, Figure S4: Calibration curves: (a) HL-60 cells and 1MPs (1:1) with regression equation $y=1.07\times 10^{-8}x$ and (b) HL-60 cells and NPs (1:1) with regression equation $y=9.55\times 10^{-9}x$, Figure S5: Focusing performance of the BAW device from Bürker counting chamber analysis and UV–Vis characterization: HL-60 cells concentrated at 2.5 × 10^6^ cells/mL mixed with 1MPs concentrated at 2.5 × 10^5^ particles/mL and HL-60 cells concentrated at 2.5 × 10^6^ cells/mL mixed with NPs concentrated at 1.8 × 10^5^ particles/mL. Mixed populations are injected at 3 µL/min when 50.59 Vpp is applied to the transducer and the device is actuated at its resonance frequency of 4.623 MHz.
