# Supplementary material for: Focalization Performance Study of a Novel Bulk Acoustic Wave Device
Source: Nanomaterials (Basel). 2021 Oct 6;11(10):2630. doi: 10.3390/nano11102630 (PMC8541289; doi:10.3390/nano11102630)
Supplement: Supplementary file 1 [file nanomaterials-11-02630-s001.zip › nanomaterials-1334662-supplementary.pdf]

Supplementary Information

Focalization Performance Study of a Novel Bulk Acoustic Wave Device

Federica Barbaresco [1,2,*], Luisa Racca [2], Luca Spigarelli [2], Matteo Cocuzza [1,3], Simone Luigi Marasso [1,3], Candido Fabrizio Pirri [1,2] and Giancarlo Canavese [1,2,*]

[1] Chilab—Materials and Microsystems Laboratory, DISAT, Politecnico di Torino, Chivasso, 10034 Turin, Italy; matteo.cocuzza@infm.polito.it (M.C.); simone.marasso@polito.it (S.L.M.); fabrizio.pirri@polito.it (C.F.P.)

[2] Department of Applied Science and Technology, Politecnico di Torino, Corso Duca degli Abruzzi 24,
10129 Turin, Italy; luisa.racca@polito.it (L.R.); luca.spigarelli@polito.it (L.S.)

[3] CNR-IMEM, Parco Area delle Scienze, 37a, 43124 Parma, Italy

* Correspondence: federica.barbaresco@polito.it (F.B.); giancarlo.canavese@polito.it (G.C.)

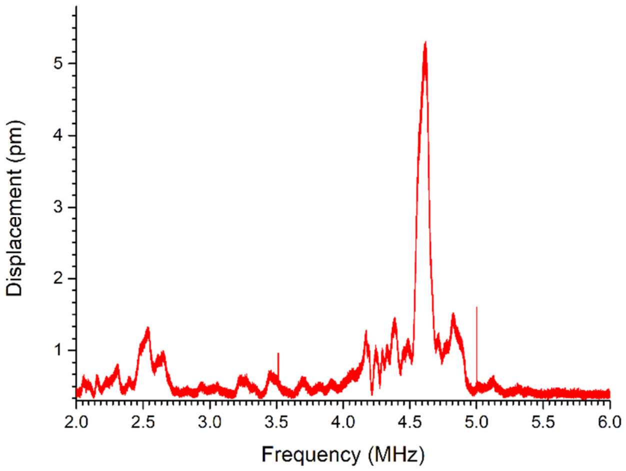

Figure S1. Measurement of the displacement of the piezoelectric element performed throughout the laser-Doppler vibrometer.

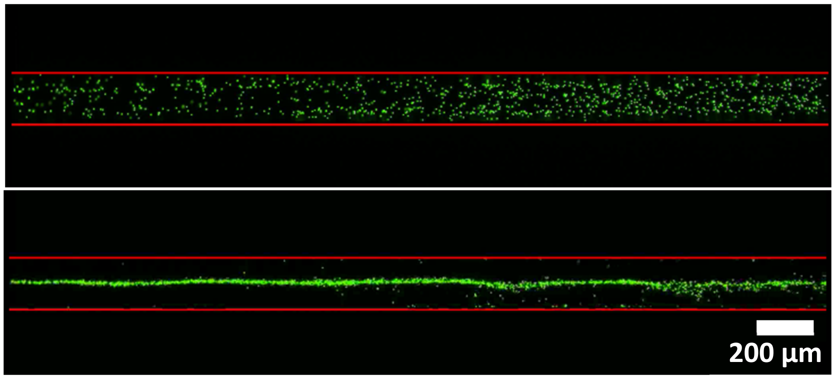

Figure S2. Frame of time-lapse acquired during the acoustophoretic test for the research of the exact resonance frequency of the BAW device. 4MPs concentrated at 5.68×10^{6} particles/mL are stable in water dispersion inside the microfluidic channel. (a) Ultrasound off and (b) Ultrasound on. Images acquired with 4x objective lens with an exposure time of 9.8 ms.

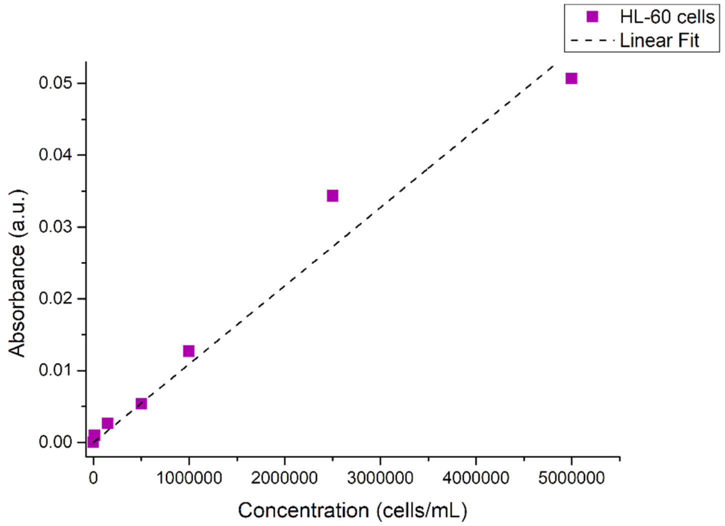

Figure S3. Calibration curve of HL-60 cells with regression equation $y = 1.09 \times 10^{-8}x$.

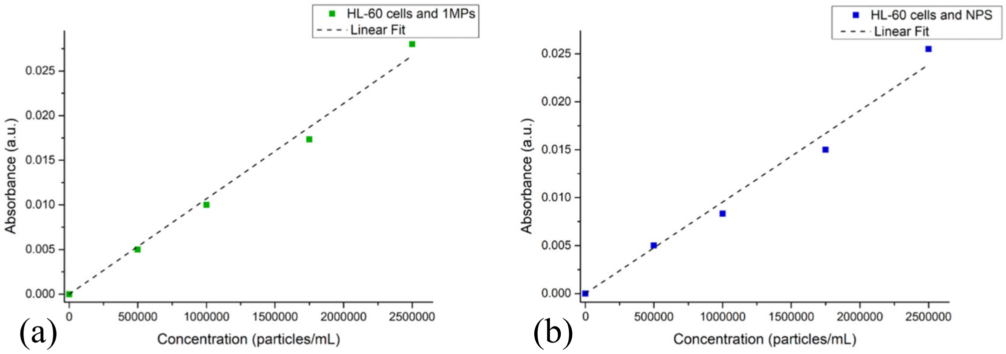

Figure S4. Calibration curves: (a) HL-60 cells and 1MPs (1:1) with regression equation $\boldsymbol{y = 1.07 \times 10^{-8}x}$ and (b) HL-60 cells and NPs (1:1) with regression equation $\boldsymbol{y = 9.55 \times 10^{-9}x}$.

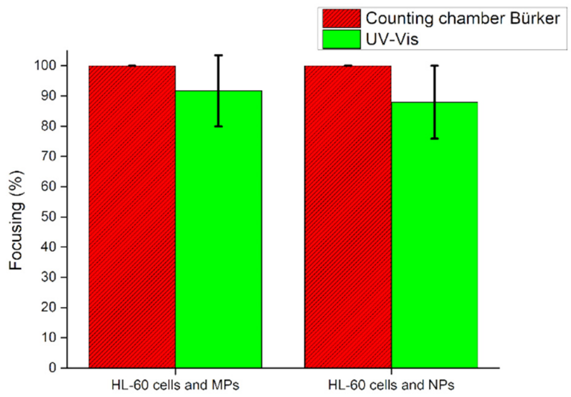

Figure S5. Focusing performance of the BAW device from Bürker counting chamber analysis and UV-Vis characterization: HL-60 cells concentrated at 2.5×10^6 cells/mL mixed with 1MPs concentrated at 2.5×10^5 particles/mL and HL-60 cells concentrated at 2.5×10^6 cells/mL mixed with NPs concentrated at 1.8×10^5 particles/mL. Mixed populations are injected at 3 μL/min when 50.59 V_{PP} is applied to the transducer and the device is actuated at its resonance frequency of 4.623 MHz.
